# Superresolution Pattern Recognition Reveals the Architectural Map of the Ciliary Transition Zone

**DOI:** 10.1038/srep14096

**Published:** 2015-09-14

**Authors:** T. Tony Yang, Jimmy Su, Won-Jing Wang, Branch Craige, George B. Witman, Meng-Fu Bryan Tsou, Jung-Chi Liao

**Affiliations:** 1 Institute of Atomic and Molecular Sciences, Academia Sinica, Taipei, 10617, Taiwan; 2 Department of Biomedical Engineering, Northwestern University, Evanston, IL 60208, USA; 3 Institute of Biochemistry and Molecular Biology, National Yang Ming University, Taipei, 11221, Taiwan; 4 Department of Cell and Developmental Biology, University of Massachusetts Medical School, Worcester, MA, 01655, USA; 5 Cell Biology Program, Memorial Sloan-Kettering Cancer Center, New York, NY 10065, USA

## Abstract

The transition zone (TZ) of primary cilia serves as a diffusion barrier to regulate ciliogenesis and receptor localization for key signaling events such as sonic hedgehog signaling. Its gating mechanism is poorly understood due to the tiny volume accommodating a large number of ciliopathy-associated molecules. Here we performed stimulated emission depletion (STED) imaging of collective samples and recreated superresolved relative localizations of eight representative species of ciliary proteins using position averages and overlapped with representative electron microscopy (EM) images, defining an architectural foundation at the ciliary base. Upon this framework, transmembrane proteins TMEM67 and TCTN2 were accumulated at the same axial level as MKS1 and RPGRIP1L, suggesting that their regulation roles for tissue-specific ciliogenesis occur at a specific level of the TZ. CEP290 is surprisingly localized at a different axial level bridging the basal body (BB) and other TZ proteins. Upon this molecular architecture, two reservoirs of intraflagellar transport (IFT) particles, correlating with phases of ciliary growth, are present: one colocalized with the transition fibers (TFs) while the other situated beyond the distal edge of the TZ. Together, our results reveal an unprecedented structural framework of the TZ, facilitating our understanding in molecular screening and assembly at the ciliary base.

Primary cilia mediate essential signaling and sensation of cells, hosting molecular activities of sonic hedgehog, non-canonical Wnt, PDGFα, and Notch signaling^1^,^2^,^3^,^4^. Dysfunctions of ciliary proteins result in serious developmental defects and can be embryonic lethal or cause pleiotropic ciliopathies including Meckel-Gruber syndrome, Joubert syndrome, nephronophthisis, and Bardet-Biedl syndrome^5^,^6^,^7^,^8^,^9^,^10^,^11^. The majority of these disorders are resulted from mutations of proteins at the TZ^5^,^12^,^13^,^14^,^15^, a region which serves both as the foundation of the cilium and as a gate for ciliary protein passage^16^,^17^,^18^,^19^,^20^.

The TZ is a specific region at the ciliary base between the BB and the axoneme proper. At the TZ, ciliary microtubules are cross-linked to the surrounding membrane through multiple rows of complex structures described, by EM, as the Y-shaped linkers (Y-links) and the ciliary necklace^21^. Several multiprotein complexes, including NPHP1–4–8^10^,^16^, MKS/B9^10^,^17^,^18^, and CEP290/NPHP5^10^,^22^ complexes, have been shown to localize to the TZ, hypothesized to compose parts of the Y-links^23^. MKS and NPHP complexes were shown to serve as elements stabilizing the Y-links and screen plasma membrane proteins^16^. RPGRIP1L was found to play a central role in anchoring other MKS and NPHP proteins^16^. RPGRIP1L binds RPGR^24^, a protein homologous to the exchange factor for Ran GTPase of nuclear pores, potentially suggesting its roles associating with GTPase at the ciliary base. CEP290 is a protein predicted to have extensive coiled-coil domains and has been shown to serve roles in tethering ciliary membranes to axonemal microtubules^22^. It interacts with several other TZ and centriolar proteins^17^,^25^,^26^,^27^,^28^,^29^,^30^,^31^,^32^,^33^. Mapping the locations of these TZ proteins, which in this study can now be reconstructed via superresolution microscopy, will greatly enhance our ability to elucidate the potential relations leading to their tethering and anchoring functions.

The entrance and exit of axonemal precursors, receptors, ion channels, and other signaling molecules are regulated at the TZ^16^,^17^,^18^,^19^,^20^, where IFT is associated with precursor and membrane protein trafficking for ciliogenesis and signaling^16^,^17^,^22^,^34^,^35^,^36^,^37^,^38^,^39^,^40^,^41^. Membrane proteins and IFT proteins pass through the TZ to reach the ciliary compartment. It remains uncertain whether these proteins travel by random diffusion through vacant spaces in the TZ or via defined paths such as pores formed by the TZ proteins^19^,^20^,^42^. Several hypotheses have been proposed as to how TZ components are architecturally organized and function with respect to the Y-links^10^,^16^,^17^,^23^,^43^. The dwell and the injection event of IFT proteins at the ciliary base have been reported^34^,^40^, and we have recently shown possible distribution patterns of IFT88 at the base of primary cilia^44^. However, the regulation mechanism and the process of IFT injection remain vague. TFs have been shown to act as the docking site for one of the IFT proteins, IFT52, potentially serving as the recruiting site for IFT particles^40^. What path these IFT proteins follow in moving from this docking site to the axoneme and how they enter the ciliary proper have never been reported. The ciliary tip is obviously a trafficking rest, exchanging anterograde IFT movement to retrograde movement, but the location of the trafficking rest at the ciliary base where retrograde to anterograde IFT exchange occurs is not known. Transmembrane proteins such as TMEM and TCTN proteins are localized at the ciliary base, with traces entering the ciliary compartment occasionally observed^17^. Where exactly the trafficking rests of transmembrane proteins at the TZ also remain elusive.

The major challenge of observing these molecules results from the tiny volume of the region close to the TZ, spanning only 250 nm in diameter and 300–1000 nm in length, packed with a large number of protein species. Conventional optical microscopes limited by the diffraction have a resolution of ~250 nm for visible light, impossible to resolve the relative positions of proteins in this region. Immuno-EM has been used to reveal the localization of various proteins at the ciliary base^22^,^40^,^45^, but the sample preparation can be challenging, and the results usually provide only a small fraction of molecules. Recent advances in superresolution microscopy have opened doors for investigations of primary cilia^46^. Among various superresolution imaging techniques, STED microscopy is solely optical physics-based with homogeneous fluorophore illumination suitable for non-filamentous protein imaging, reaching 50-nm resolution for cell imaging^47^,^48^,^49^. 3D superresolution microscopy techniques have been developed to obtain subdiffraction images of molecules in all three dimensions^50^,^51^. Nevertheless, we will show that the characteristics of the point spread function (PSF) of 2D STED microscopy make it simpler than 3D superresolution methods for revealing diameters of radially symmetric ciliary proteins.

The present study aimed to reveal the architectural organization of the ciliary base beyond the diffraction limit. We used STED microscopy to create a molecular map of important proteins in the TZ, the TFs, and the BB. This mapping was made possible through pattern recognition of superresolution images resulting from convolving our specific STED PSF with signals of ring-shaped proteins. Multiple STED images were overlapped with EM images to reveal the molecular architecture at the ciliary base. Upon this molecular architecture, we found two levels of trafficking rests for IFT proteins. We also found that the distributions of IFT proteins were dependent on ciliary growth conditions, suggesting a mechanism for entrance regulation at the base of primary cilia. In addition, we identified accumulation sites of transmembrane proteins at a level different from the two for IFT proteins. Together, this study revealed distinguished levels of the molecular organization and trafficking rests at the ciliary base, shedding light on the gating mechanism of the passage barrier at the TZ.

## Results

### The unique point spread function of STED facilitates pattern recognition of ring-shaped protein distributions

To localize various TZ proteins, many of which presumably exhibit annular symmetry in relation to the axoneme, we used the slender PSF of a 2D STED system, taking advantage of its relative ease in determining radial distributions of ring-shaped proteins. Although aligning the light path of the objective with the axonemal axis of a cilium seems proper to visualize radially distributed TZ proteins, signals from different planes along the cilium challenged the identification of the plane of interest, making it difficult to determine their localizations. In contrast, a lateral view with the light path perpendicular to the axonemal axis gave a high success rate in observing radial allocations of ring-shaped protein distributions despite signals from off-focus parts of the ring. The long and slender PSF of 2D STED with ~50–60 nm lateral full width at half maximum (FWHM) and ~450 nm axial FWHM, potentially unfavorable for resolving particles in the z-direction, actually augmented signals from the periphery of a ring due to high density projection. As shown in the simulation results (Supplementary Fig. 1a,b), when using a PSF of 2D STED, the ring-shaped pattern becomes a rectangular-like pattern with similar side-to-side distance for different focal planes. That is, the image would still give a two-peak pattern with peak-to-peak distance varying insignificantly up to ~±300 nm of mis-focusing (Supplementary Fig. 1c). On the contrary, when a more sophisticated 3D STED technique is used^52^, the signals would follow the ring pattern closely, causing the variation in diameter to be dependent upon the focal plane (Supplementary Fig. 1d–f). Knowing the expected patterns of rings of different diameters, we searched for these patterns when imaging different ciliary proteins. Repeated appearances of similar patterns for a specific protein were collected to statistically determine the diameter of each protein.

### TZ proteins CEP290, RPGRIP1L, MKS1, TCTN2, and TMEM67 allocate differentially in width

**Figure 1:**
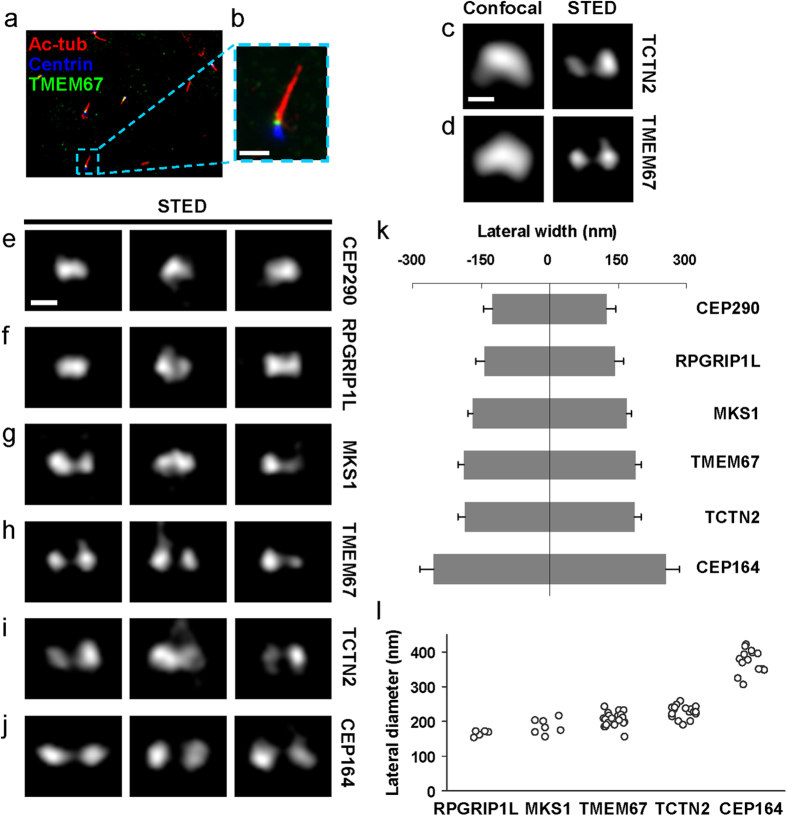
Subdiffraction STED images revealing distinct lateral localization patterns of TZ/TF proteins at the base of primary cilia in RPE-1 cells. (**a**,**b**) An epifluorescence image of RPE-1 cells showing the TZ protein TMEM67 (green) sandwiched between centrin-eGFP^60^ (blue) and acetylated tubulin (red). Scale bar: 2 μm. (**c**,**d**) Comparison of confocal and STED images of TCTN2 and TMEM67 illustrating the ability of STED to resolve two separate intensity peaks. (**e**,**j**) A set of STED images showing distinct lateral dimensions of TZ and TF proteins. CEP290 and RPGRIP1L had narrow widths, while MKS1, TMEM67, TCTN2, and CEP164 had separable intensity peaks in the order of increasing widths. Scale bar, 200 nm. (**k**) Lateral widths (mean ± standard deviation) of TZ/TF proteins. (**l**) Distributions of lateral diameters of TZ/TF proteins defined by lateral distances between two intensity peaks.

Human retinal pigment epithelial cells (RPE-1) were chosen for these studies as they frequently have primary cilia aligned parallel to the imaging plane (Supplementary Fig. 2), allowing simultaneous mapping of proteins at the ciliary base in both the radial and axial directions (Fig. 1a,b). To relate known TZ structures to specific functional protein complexes, selected components known to be associated with the three major multiprotein complexes of the TZ were imaged, including CEP290, RPGRIP1L, MKS1, TMEM67, and TCTN2. In addition, CEP164, a component of the TFs known to mediate the anchorage of BBs to the plasma membrane, was examined to establish the proximal end of the TZ.

We searched for patterns of two separate intensity peaks for several TZ proteins in superresolution images (Fig. 1c,d). The radial distribution of TZ proteins around the axoneme can be directly inferred from the shape and size of the signal. CEP290 consistently displayed an elliptical- or rectangular-like shape (Fig. 1e), with a width similar to that of the axoneme. RPGRIP1L occupied a similar width as CEP290 and the axoneme, with some images showing two intensity peaks (Fig. 1f, middle panel). Subdiffraction imaging of MKS1 (Fig. 1g), TMEM67 (Fig. 1h), and TCTN2 (Fig. 1i) showed that they occupied a wider area, many clearly resolved into two separate intensity peaks, indicating that they were localized toward the outer periphery of the TZ. Note that in some of the TMEM67 and TCTN2 images (Fig. 1h,i), branches into the ciliary proper from one or both of the intensity peaks were observed, consistent with previous observation^17^, implying that TMEM67 and TCTN2 may be able to move dynamically instead of being fixed parts of the ciliary structure. Subdiffraction imaging of CEP164 revealed a wider lateral occupation than any TZ protein examined in this study (Fig. 1j), marking the positions of TFs^53^. Analyses of the lateral widths defined by the FWHMs and of the lateral diameters defined by the lateral distances between two intensity peaks (Supplementary Fig. 3) revealed that TZ components occupy different regions of the TZ, with CEP290 and RPGRIP1L being the narrowest, TMEM67 and TCTN2 being the widest, and MKS1 being intermediate in width (Fig. 1k,l and Supplementary Table 1). Note that these differential allocations would not have been distinguished without superresolution microscopy.

### Transmembrane proteins aggregate at the same axial level as that of MKS1 and RPGRIP1L but different from the level of CEP290

**Figure 2:**
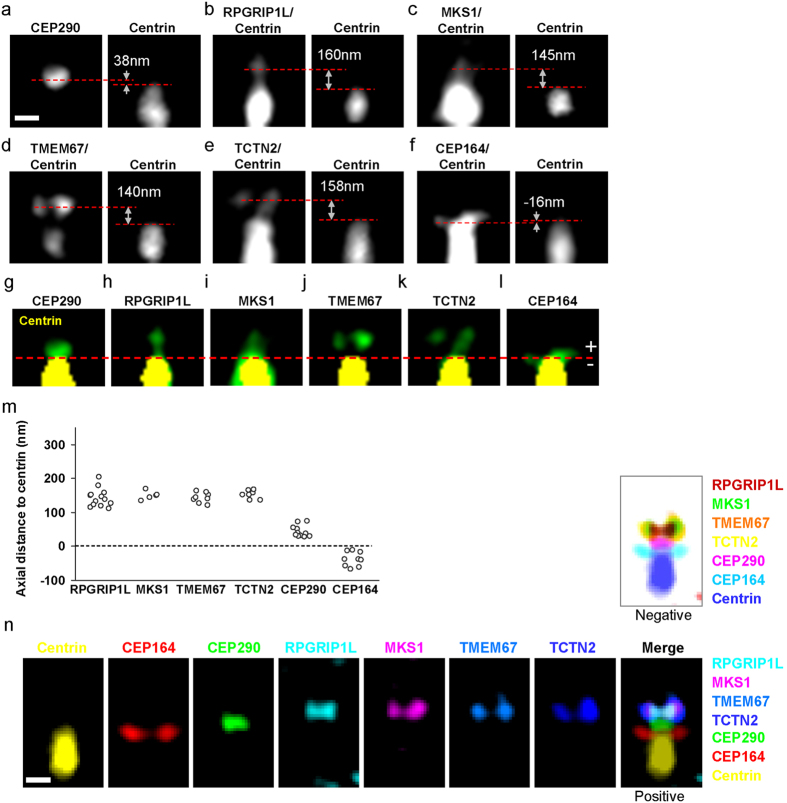
Dual-color STED images revealing distinct axial localization levels of different TZ and TF proteins. (**a**–**f**) Sample dual-color STED images showing different axial positions relative to the distal edge of centrin for different TZ/TF proteins. The axial distance of each BD V500-labeled TZ/TF protein to the FWHM-defined boundary of centrin-eGFP was measured. Centrin-eGFP signal could also be seen in the BD V500 channel. (**g**–**l**) Comparison of the axial positions of TZ/TF proteins (green) relative to centrin (yellow) revealing that TMEM67 and TCTN2 were dwelled at a similar axial level to MKS1 and RPGRIP1L in terms of the distance to centrin (red dashed line), while CEP290 was at another axial level close to centrin, distinct from the other TZ proteins. CEP164 was slightly proximal to the distal edge of centrin. Scale bar for (**a**–**l**): 200 nm. (**m**) Comparison of the axial distances to centrin. RPGRIP1L, MKS1, TMEM67, and TCTN2 were ~150 nm from the centrin edge, while CEP290 was ~40 nm from the centrin edge. (**n**) A series of single-color STED images of TZ/TF proteins were axially positioned based on the average relative distances to the distal edge of centrin from dual-color STED images. The threshold of each single-color image was adjusted based on the FWHM to represent their shape and size. These axially-positioned images were overlapped to create a 7-color superresolution image of proteins at the ciliary base. To obtain a white-background image to be merged with an EM image, colors of the merged multi-color image were inverted with a corresponding negative mapping (upper). Scale bar: 200 nm.

To reveal the relative localization of ciliary proteins, dual color STED superresolution imaging was performed using centrin 2-eGFP (491 nm excitation) and antibody stained TZ/TF proteins with organic dye BD V500 (447 nm excitation). Centrin 2 in the BB is a reliable reference coordinate because the axial distances between the “distal edge” of centrin and the TZ proteins are large enough to have minimal signal overlap and, most importantly, because all measurements were reproducible with small variations (see Supplementary Table 1). We revealed differential axial arrangement of CEP290, RPGRIP1L, MKS1, TMEM67, TCTN2, and CEP164 (Fig. 2a–f and Supplementary Fig. 4). The axial distance from the location of highest intensity for a specific protein to the distal tip of centrin 2 was recorded. The average distances for CEP290, RPGRIP1L, MKS1, TMEM67, TCTN2, and CEP164 were 42 ± 16 nm, 143 ± 27 nm, 150 ± 12 nm, 143 ± 15 nm, 153 ± 13 nm, and −38 ± 21 nm, respectively, with the ciliary tip as the plus direction and the BB as the minus direction (Supplementary Table 1). A representative set of side-by-side dual-color STED images with aligned distal edges of centrin are shown in Fig. 2g–l. Markedly, CEP290 is localized at a distinct axial level situated between the BB protein centrin 2 and other TZ proteins (Fig. 2m). The intensity peak of the TF protein CEP164 was proximal to the distal edge of centrin (Fig. 2l,m). The highest intensity points of TMEM67 and TCTN2 occurred at the same axial level as MKS1 and RPGRIP1L, indicating a special axial level accommodating these ciliopathy-associated proteins.

### Overlapping multiple superresolution images with EM images recreate spatial registration of ciliary proteins upon the TZ ultrastructure

**Figure 3:**
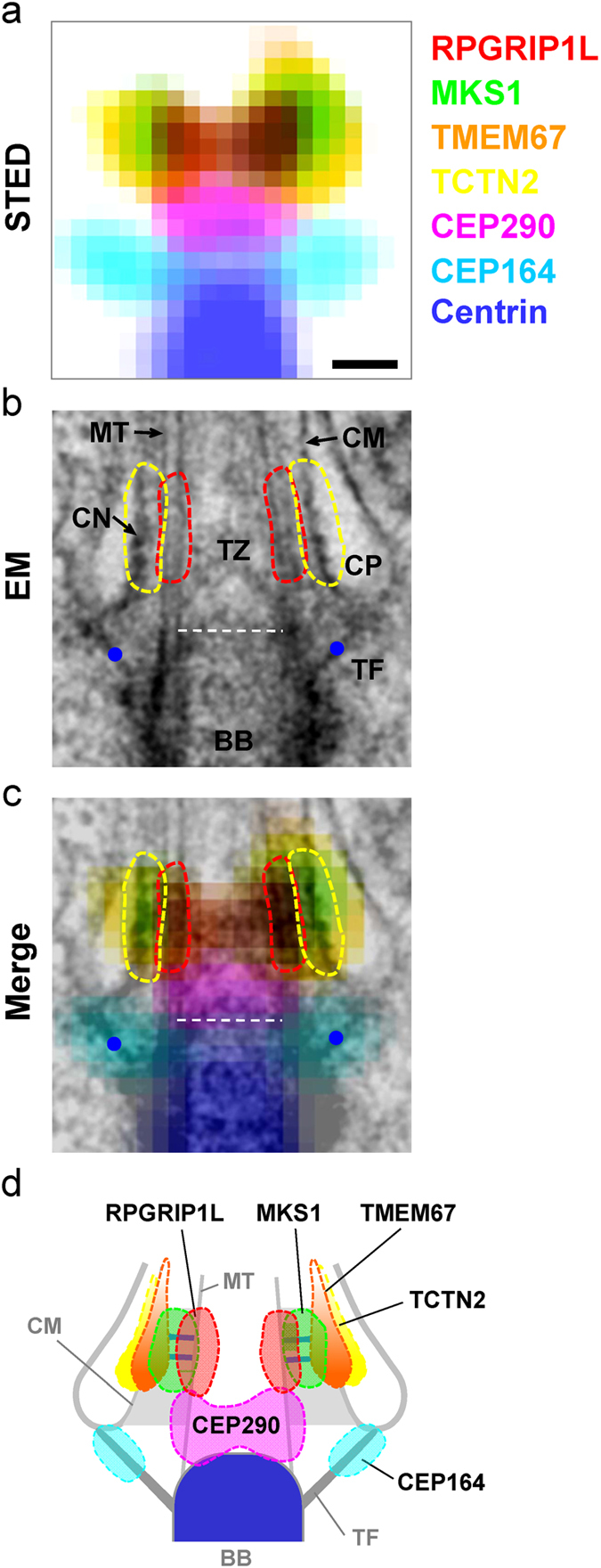
Molecular architecture at the base of the primary cilium obtained by overlapping coordinate-defined superresolution images of TZ/TF proteins and an EM image. (**a**) A 7-color superresolution image obtained by merging multiple single-colored STED images illustrating the relative locations of important TZ/TF proteins. (**b**) A typical equally-magnified EM image of a primary cilium in an RPE-1 cell, where TFs are marked as blue dots, areas of microtubule doublets (MT) circled with red dashed lines, areas of the ciliary membrane (CM) covering the ciliary necklace (CN) circled with yellow dashed lines, and the distal end of the BB marked by a white dashed line. CP: ciliary pocket. (**c**) A merged image of (**a**,**b**) obtained by aligning the TFs in EM and CEP164 in STED. RPGRIP1L was close to the microtubule doublets; TMEM67 and TCTN2 were localized mostly toward the TZ membrane, while MKS1 was localized midway between the membrane and microtubules. CEP290, right above the BB, was localized at a different axial level from the other TZ proteins. Scale bar, 200 nm. (**d**) A localization model of TZ/TF proteins at the ciliary base pinpointing the positions of these proteins relative to each other and to known structural elements. Y-links are hypothesized to bridge across the level where RPGRIP1L and MKS1 are occupied, with dwelling transmembrane proteins such as TMEM67 and TCTN2 decorated as the ciliary necklace.

To generate an architectural map of the ciliary base, subdiffraction images of individual proteins from single-color STED microscopy were overlapped, based on their average positions relative to centrin, to create a multicolor superresolution image (Figs 3a and 2n). This 7-color superresolution image (Fig. 3a) was then overlapped with EM images of longitudinal sections through the bases of primary cilia of RPE-1 cells (Fig. 3b and Supplementary Fig. 5) by placing the intensity peaks for CEP164 on top of the TFs clearly seen in the EM images (Fig. 3c). From the merged EM-STED images (Fig. 3c and Supplementary Fig. 5C,F), RPGRIP1L, MKS1, TMEM67, and TCTN2 can be seen at the axial level close to the arrays of puncta on the ciliary membrane on one side of the ciliary pockets shown in EM, presumably the ciliary necklace. Radially, RPGRIP1L was localized near the TZ microtubules; TMEM67 and TCTN2 were close to the TZ membrane with their high intensity puncta covering the region of the ciliary necklace with occasional leakage signals branching toward the ciliary compartment; and MKS1 is located between RPGRIP1L and the high intensity puncta of TMEM67/TCTN2 (Fig. 3d). CEP290 was localized to a narrow region of the TZ between the BB distal end and the other TZ proteins, with a radial width similar to that of the axoneme; this pattern was confirmed using two distinct antibodies to CEP290 (Supplementary Fig. 6). The results suggest that the coiled-coil protein CEP290 serves its functional or structural roles at a level distinct from other TZ proteins, while GTPase-associated RPGRIP1L is placed toward the axoneme for its potential interaction with RPGR. TMEM67 and TCTN2 tend to dwell at the level of MKS1 and RPGRIP1L, illustrating the preferred resting site for these portable transmembrane proteins. Together, we recreated the spatial registration of TZ proteins upon a basic architectural map of the ciliary base.

### IFT particles rest at two distinct axial levels, one at the TFs and the other beyond the distal edge of the TZ

**Figure 4:**
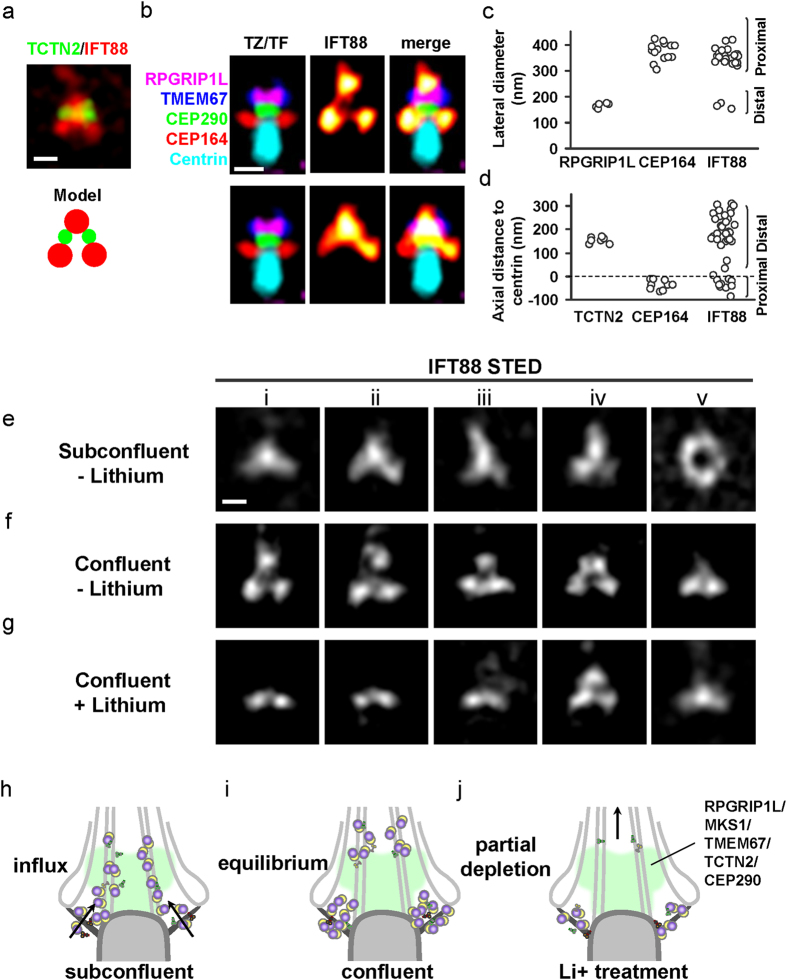
Effects of ciliary growth conditions on the distribution patterns of IFT88 at the ciliary base. (**a**) A dual-color STED image and a proposed model of TCTN2 and IFT88 showing that TCTN2 was localized axially between the distal and proximal IFT88 puncta. (**b**) Three-puncta (upper panels) and Y-shaped (lower panels) patterns of IFT88 superimposed on the TZ/TF framework. IFT88 was localized at two levels, one at the TF and the other close to the distal edge of the TZ. (**c**,**d**) Similar lateral and axial positions of CEP164 and the proximal puncta of IFT88 suggested that IFT88 is localized to TFs. (**e**) Sample STED images of IFT88 at the ciliary base of subconfluent RPE-1 cells (~700 cells/mm^2^) exhibiting a dominant population of the Y-shaped pattern. Sometimes an IFT88 aggregate was found to possess a full-ring pattern. (**f**) A dominant population of the three-puncta distribution pattern of IFT88 was observed for confluent cells (~1000 cells/mm^2^). (**g**) A novel two-puncta pattern of IFT88 prevailed at the base of cilia stimulated to elongate by 100 mM Li^+^; Li^+^ treatment depleted the IFT88 population at the distal punctum within an hour. Scale bar for (**a**,**b**,**e**–**g**), 250 nm. (**h**–**j**) Models depicting postulated movements of IFT88 consistent with the protein’s three different distribution patterns.

Using this architectural map as a guide, we next sought to understand how IFT particles travel through the ciliary base. We observed two distinct distributions of IFT particles at the ciliary base in RPE-1 cells, namely the Y-shaped and three-puncta patterns, corresponding to those we previously observed in human fibroblasts^44^. To map these patterns upon the molecular architecture, we performed dual-color STED imaging of IFT88 and TCTN2 (Fig. 4a). For the three-puncta pattern of IFT88, the proximal and distal puncta were located immediately proximal and distal to the TZ proteins in the axial direction, with the proximal puncta colocalized with the TF component CEP164 and the distal punctum situated mostly at the distal end of TCTN2 beyond the distal edge of the TZ (Fig. 4b–d, upper). This suggests that there are two levels of IFT88 accumulation, one at the TF and the other beyond the distal edge of the TZ, representing two levels of trafficking rests for IFT particles. The distal punctum occupied a similar width as CEP290 and RPGRIP1L, with some images showing two intensity peaks, potentially localized to the axoneme beyond the TZ. For the Y-shaped pattern, the distal branch of the Y spread towards the ciliary compartment, while the two proximal branches were localized at the proximal edge of the TZ, extending toward CEP164 (Fig. 4b, lower). Thus, IFT88 decorated across the TZ with a tilted angle from the TFs to the TZ, suggesting defined trafficking paths of IFT particles at the ciliary base.

### Perturbation of ciliary growth significantly transforms distribution patterns of IFT particles at the ciliary base

To further understand the functional relevance of these two distribution patterns of IFT particles, we tested different perturbation effects on these patterns. Intriguingly, distribution of IFT88 was correlated with specific growth conditions of cilia (Fig. 4e–g). The Y-shaped pattern was predominately associated with growing or elongating cilia in a sub-confluent culture (~700 cells/mm^2^) (Fig. 4e and Supplementary Fig. 7A), whereas the three-puncta pattern was mostly seen in fully elongated cilia of a confluent culture (~1000 cells/mm^2^) (Fig. 4f). Moreover, when a confluent culture was treated with lithium ion^54^ for one hour to induce excess elongation of cilia (4.3 ± 0.8 μm with Li^+^ vs 3.6 ± 0.8 μm without Li^+^), the IFT88 distribution at the ciliary base quickly changed from a 3-puncta pattern to a 2-puncta pattern with the distal punctum mostly depleted (Fig. 4g), coinciding with marked accumulation of IFT88 at the ciliary tip (Supplementary Fig. 7B). Together, these distinct patterns of IFT88 reflect the movement of IFT particles through the ciliary gate in response to different phases of ciliary growth. Ongoing ciliary growth resulted in continuous spreading of IFT88 across the TZ, while mature ciliogenesis resulted in accumulation of IFT88 at two distinct axial levels at the ciliary base. Excess elongation of cilia nearly exhausted IFT88 at the distal edge of the TZ within one hour, reflecting a dynamic change of IFT trafficking made visible by superresolution imaging.

## Discussion

Here we have revealed the superresolved architectural organization at the base of primary cilia. We determined that there are at least two layers of proteins at the TZ: one containing RPGRIP1L and MKS1 and the other containing CEP290. Transmembrane proteins TMEM67 and TCTN2 concentrate at a space corresponding to the ciliary necklace, which decorates the ciliary membrane at the same axial level as RPGRIP1L and MKS1, though TMEM67 and TCTN2 can also spread into the ciliary proper. The molecular structure at the axial level of MKS1 and RPGRIP1L potentially forms a resting or recruiting site for transmembrane proteins TMEM67 and TCTN2. IFT88 distributes across the TZ with different patterns depending on the phase of ciliary growth. These patterns consist of at least two levels of trafficking rests: one at the TFs and the other beyond the distal edge of the TZ distinct from the level at which TMEM67 and TCTN2 localize. The sites of IFT particle accumulation likely represent important zones for cargo loading, processing, or sorting of the IFT machinery.

The architectural organization of TZ components at the ciliary base reveals their spatial relationships not only with each other, but also with the TFs, the TZ membrane, the axoneme, and the dynamic sites of IFT particle accumulation. In this map, CEP290 forms the base of the TZ, upon which TCTN2, TMEM67, MKS1, and RPGRIP1L assemble and organize to occupy the space between the axoneme and membrane, where they would be ideally located to function as part of the diffusion barrier. RPGRIP1L is near the TZ microtubules, TCTN2 and TMEM67 are at the membrane, and MKS1 appears to be located in between RPGRIP1L and TCTN2/TMEM67 (Fig. 3d). It is possible that these proteins are involved in the formation of Y-links bridging the ciliary membrane and the axoneme, some playing anchoring or regulatory roles while others serving as structural elements of the Y-links. Future structural and functional studies of the TZ guided by this architectural map will enhance our understanding of the biogenesis and function of cilia.

The advent of superresolution techniques opens up access to novel observation at the subdiffraction scale, particularly suitable for studies of primary cilia. The usage of the slender PSF of 2D STED for our problem was especially unique in its convolving effect on ring-shaped signals. As we know that all superresolution microscopy techniques have inherent challenges, this effect significantly reduced the difficulty of imaging TZ proteins, allowing us to focus our efforts on searching for patterns, which still required multi-focal scanning and large area lateral browsing. Thus, we were able to repeatedly recognize expected patterns to collect enough images for statistical analysis of TZ protein localizations.

One general concern for superresolution immunofluorescence imaging is the spatial extent of antibodies. Primary and secondary IgG antibodies together introduce ~16 nm uncertainty to the protein localization (detailed information of the primary antibodies utilized in these studies is shown in Supplementary Table 2). This extent is smaller than the STED optical resolution (>50–60 nm). Furthermore, the peak-to-peak distances reported in this work were based on the ensemble measurement and not by single molecule information, so the distance error of indirect labeling is alleviated by averaging. Taking these two factors into consideration, the localization error caused by indirect immunofluorescence was acceptable at our resolution limit.

It is known that morphological characteristics of primary cilia such as the ciliary length are cell-type dependent. Thus, the molecular distributions at the TZ can also be cell-type or cell-line dependent, although it is expected that its major structural components may be similar among different mammalian cell lines. This study utilized RPE-1 cells for the first TZ mapping. As illustrated in Supplementary Fig. 2, RPE-1 cells were chosen because their primary cilia more consistently aligned parallel to the plane of imaging. Further superresolution studies of other cell types will be needed to validate the applicability of the results found in this work and to reveal novel cell-type dependence of TZ architecture.

Our data show that RPGRIP1L, MKS1, TCTN2, and TMEM67 are localized at a similar axial level with different radial positions, spanning from the axoneme to the ciliary membrane. A previous study showed that mutated MKS5 (a RPGRIP1L homolog) in *C. elegans* caused serious structural defects of its sensory cilia, anchoring aberrations of other TZ proteins, and gating leakages^16^,^55^. As we found that RPGRIP1L is localized toward the axoneme, it is possible that it anchors the microtubule-membrane linkers at the axonemal end, and thus dysfunction of the protein destroys the TZ structure and results in structural and functional aberrations. Since RPGRIP1L is associated with proteins homologous to those involved in nuclear pore barrier formation^24^, this localization is likely to be mechanistically significant for ciliary gating, as suggested by the similarity to nuclear pores^19^. Previous data showed that mutation of MKS1 did not affect cilium morphology in *C. elegans* unless combined with a mutant of NPHP1 or NPHP4^16^, despite that MKS1 mutation alone is known to be associated with the serious mammalian MKS^56^. Our observations showed that MKS1 is localized between the ciliary membrane and the axoneme. A rational hypothesis is that MKS1 and NPHP proteins serve as complementary components of the bridge between the ciliary membrane and axoneme, possibly as structural components of the Y-links^21^. Our observation that TCTN2 and TMEM67 are localized toward the ciliary membrane is consistent with their known membrane affinity^10^,^16^,^17^,^57^, possibly related to the necklace intramembrane particles^21^. It is known that mutation of TCTN2 affects ciliogenesis in a tissue-dependent manner, while mutation of TMEM67 has slightly different ciliogenic defects from those of TCTN2^10^,^17^. Their localizations at the same level as MKS1 and RPGRIP1L imply that they may interact directly with the proteins at this level, possibly serving regulatory roles for tissue-specific ciliogenesis. The observation of a small population of them in the ciliary proper indicates that they may travel along the ciliary membrane across the TZ, possibly interacting with other membrane-bound proteins in the ciliary compartment. This localization can be important in understanding GPCR translocation through the barrier. Based on our findings, CEP290 is located in the proximal TZ, potentially serving as a foundation for TZ formation. Although the finding of CEP290’s localization is novel, it is consistent with previous data indicating that CEP290 serves a tethering role in forming microtubule-membrane linkers and maintaining normal protein composition^22^, because the loss of CEP290 may affect the positioning of all other TZ proteins relative to the distal end of the BB.

The distribution patterns of IFT88 clearly showed two levels of IFT-particle trafficking rests at the ciliary base, one at the TFs and the other beyond the distal edge of the TZ. Localization at the TFs presumably suggests assembly of the IFT particles at the TFs prior to entering the ciliary proper. A hypothesis stated below is consistent with the observed patterns in different ciliary growth conditions. When cells were in a subconfluent condition, IFT particles likely undergo continuous entry into the ciliary compartment to form influx streams through the TZ; these streams of IFT particles are imaged as the two branches of the Y-shaped pattern (Fig. 4h). Newly formed IFTs are continuously entering the cilium and are often captured in the TZ, perhaps reflecting slower passage through this zone than through the ciliary proper. When cilia reach their full length under confluent conditions, the system reaches equilibrium whereby IFT particles accumulate in two reservoirs corresponding to the proximal puncta of the three-puncta pattern at the TFs and the distal punctum on or at the distal side of the TZ (Fig. 4i). Stimulation of further ciliary growth following addition of Li^+^ quickly depletes IFT particles at the distal resting site to deliver additional axonemal proteins toward the ciliary tip, leaving a two-puncta pattern at the TFs (Fig. 4j). The resting site at the TF level implies that the TFs may act as the recruiting site for IFT particles in the cytosol, while the resting site beyond the distal edge of the TZ may serve as a “bus stop” or an “assembly factory” for anterograde and retrograde trafficking as well as selective signaling activation. Localized proteins at these levels, especially those with regulatory functions such as kinases TTBK2 and NEK8^58^,^59^, can also be further explored to gain a greater understanding of the underlying reasons of hosting these specific resting sites.

## Methods

### Cell Culture

Human RPE-1 cells (in some cases with stably expressed centrin 2-eGFP)^60^ were grown on #1.5 cover glasses coated with poly-L-lysine, in DMEM/F12 supplemented with 10% FBS, 12 mM HEPES, 2.5 mM L-glutamine, 2.4 g/L sodium bicarbonate, and 1% penicillin/streptomycin at 37 °C and 5% CO_2_ up to 60% ∼ 90% confluence depending on the experimental conditions, and then incubated in serum-deprived media for 48 hours. For lithium stimulation experiments, cells were treated with 100 mM lithium chloride (L121-100, Fisherbrand) for one hour before fixation.

### Antibodies

Ac-tub antibody (anti-acetylated α-tubulin mouse IgG, ab24610, Abcam) was used as a ciliary marker at 1/1000 dilution for epifluorescence imaging and 1/2000 for single-color STED imaging. IFT88 (anti-IFT88 rabbit IgG, 13967-1-AP, Proteintech) was prepared at 1/200 dilution. RPGRIP1L (anti-RPGRIP1L rabbit, HPA039405, Sigma-Aldrich, at 1/300 dilution), TCTN2 (anti-TCTN2 mouse IgG2a, ab119091, Abcam, at 1/500 dilution), MKS1 (anti-BBS13 rabbit IgG, 16206-1-AP, Proteintech, at 1/50 dilution) and TMEM67 antibodies (anti-TEME67 rabbit IgG, 13975-1-AP, Proteintech, at 1/200 dilution) were used for labeling the TZ. For CEP290, two antibodies were used, one for the N-terminal and one for the C-terminal (see comparison in Supplementary Fig. 6). For the N-terminal CEP290 antibody, a synthetic peptide consisting of residues 11–24 of mouse Cep290 with a C-terminal cysteine added for conjugation to KLH (IKVDPDDLPRQEEL-C) was used to generate rabbit polyclonal antibodies (Genscript). The resulting antiserum was affinity purified on an agarose support (Thermo Fisher Scientific) according to the manufacturer’s instructions. This N-terminal CEP290 antibody was used at 1/100 dilution. The C-terminal CEP290 (anti-CEP290 rabbit IgG, ab84870, Abcam) against residues 2429–2479 was used at 1/500 dilution. CEP164 (anti-CEP164 rabbit, 45330002, Novus Biologicals) antibody was used at 1/2500 dilution for staining of TFs. Information of isotopes and immunogens of primary antibodies is listed in Supplementary Table 2. For secondary antibodies, Oregon Green 488 antibodies (goat anti-rabbit or goat anti-mouse, Invitrogen) were used at 1/200 dilution for TCTN2, MKS1, and RPGRIP1L and 1/1000 for TMEM67, CEP290, CEP164, and IFT88. Biotin antibodies (anti-mouse IgG, B6649 or anti-rabbit IgG, B8895, Sigma-Aldrich) were diluted at 1/200 and V500 streptavidin (561419, BD Horizon) was diluted at 1/100 for labeling the TZ/TF/IFT proteins for two-color STED imaging or at 1/500 for ac-tub and IFT88 for single-color STED imaging.

### Immunofluorescence

The cells were fixed for 5 min at room temperature with 4% paraformaldehyde (PFA) immediately followed by methanol at −20 °C for 10 min. Following fixation, cells were washed 2–3 times in PBS, permeabilized with 0.1% PBST (PBS + Triton X-100), and then blocked with 1% normal goat serum and 2.5% BSA in 0.1% PBST for 30 min at room temperature. Samples were incubated with primary antibodies in block solution for 1 hour at room temperature, and rinsed at least 3 times in PBST. Cover glasses were then incubated with secondary antibodies or biotin antibodies for 45 min at room temperature, and washed again 3 times in PBST. For biotin antibodies, the cells were stained with streptavidin conjugated to V500 dye (BD Horizon) for 20 ∼ 30 min, followed by at least 5 times in PBST. Finally, the samples were mounted with 86% glycerol in PBS.

### STED Microscopy

The home-built continuous-wave STED setup (491-nm for excitation and 592-nm for depletion) was as previously described^44^, with the addition of a 447-nm diode excitation laser (PGL-V-H-447, CNI) for dual-color STED imaging. Three laser beams were merged and focused onto the sample through a 100× oil immersion objective (Olympus UPLSAPO100×−1.4 NA). The fluorescent signals were collected with the same objective and detected by an avalanche photodiode (APD) module (SPCM-AQR-15, PerkinElmer Optoelectronics). For single-color STED imaging of TZ/TF proteins (except TCTN2), ac-tub imaged in the confocal mode (447-nm laser) was used to find cilia only and one TZ/TF protein at a time was later imaged in the STED mode (491-nm laser). Ac-tub was stained with V500 at a low concentration to avoid crosstalk to the channel of TZ/TF proteins labeled with Oregon Green 488 and illuminated at 491 nm for STED imaging. For TCTN2, anti-IFT88 detected with a low concentration of V500 was used as a ciliary marker. For dual-color STED imaging, centrin-eGFP excited at 491 nm was used to identify the ciliary base, while CEP290, RPGRIP1L, MKS1, TMEM67, TCTN2, or CEP164 was labeled with V500 at the 447-nm channel. Since GFP was partially excitable at 447 nm, centrin could be detected in both channels; as a result, two sequential acquisitions of STED images could be readily aligned by correlating the centrin location. To determine the position of IFT88 relative to TCTN2, IFT88 (447-nm laser channel) was first used to locate cilia in the confocal mode and then STED images were acquired for both proteins. For all STED imaging, the excitation power was operated at 0.3–1.5 μW depending on the signal level of TZ/TF/BB/IFT proteins. For CEP290, TMEM67, CEP164, centrin, or IFT88 where the signals were strong, the excitation laser was set close to the lower bound of the working power. For MKS1 where the signals were substantially weaker (Supplementary Fig. 8), the excitation power was generally operated at the upper bound. A medium excitation power close to 1 μW was used for imaging TCTN2 and RPGRIP1L. The power of the depletion laser was set differently for different proteins based on photon counts. The power was generally set to ~100 mW for centrin, 90 ∼ 100 mW for CEP290, TMEM67, CEP164, or IFT88, 70 ∼ 90 mW for TCTN2 and RPGRIP1L, and ~60 mW for MKS1. When signals were weakened after a few acquisition scans, the depletion power could be reduced by ~10 mW to maintain a sufficient signal level. To find an optimal focus, STED images were acquired at different planes every 100 nm around an initial position until the sample was photobleached substantially, usually after 3–5 times of scanning. For all STED scans, a step size of 25 nm and a dwell time of 20 μs were used to gain the maximal signal-to-noise ratio as well as to satisfy the Nyquist criteria for spatial sampling. The registered photon count per 20 μs was generally above 100 for confocal imaging and about 50% reduced during STED acquisition. For image display in figures, we performed deconvolution to enhance the signals and thresholding to enhance contrast relative to the background signals.

### Transmission Electron Microscopy

G1-arrested RPE-1 cells grown on coverslips made of Aclar film (Electron Microscopy Sciences) were fixed in 4% paraformaldehyde and 2.5% glutaraldehyde with 0.1% tannic acid in 0.1 M sodium cacodylate buffer at room temperature for 30 min, postfixed in 1% OsO_4_ in sodium cacodylate buffer for 30 min on ice, dehydrated in a graded series of ethanol, infiltrated with EPON812 resin (Electron Microcopy Sciences), and then embedded in the resin. Serial sections (~90 nm thickness) were cut on a microtome (Ultracut UC6; Leica) and stained with 1% uranyl acetate as well as 1% lead citrate. Samples were examined on a JOEL transmission electron microscope.

### Data Analysis

The illustrated confocal and STED images were smoothened and contrast-enhanced with generalized Tikhonov regularization^61^. Thresholding was applied to illustrate the FWHM of objects in Fig. 1c–j using ImageJ. For quantitative width measurement of the TZ/TF proteins, the STED images were first cleaned by a mean filter of 1 pixel and background subtracted. For CEP290 and RPGRIP1L, images were fitted with a two-dimensional Gaussian function 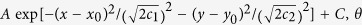 (Supplementary Fig. 3M,N) to identify the principal axes and to find the FWHMs (Supplementary Fig. 3A,B,G,H), where c1 and c2 were used to determine the FWHM by multiplying the factor of 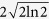. The fitting curves agreed well with the data, except that some RPGRIP1L images had a slight intensity dip in the middle. For MKS1, TMEM67, TCTN2, and CEP164, STED images clearly displayed two distinct peaks in the lateral direction, so the lateral FWHMs were determined by measuring the distances of two intensity peaks (Supplementary Fig. 3C–F,I–L). To define the reference coordinate in the axial direction, the distal edge of centrin was located by pinpointing the position of the FWHM of the fluorescent intensity along the primary axis of centrin (Supplementary Fig. 4). Note that the visual artifact of the size difference between Figs 1 and 2 (e.g. RPGRIP1L in Figs 1f and 2b; MKS1 in Figs 1g and 2c) was primarily caused by the intensity threshold chosen to show more of centrin; the measured FWHMs of these two figures were very similar and the axial distance was not affected by its visual size. To obtain the 7-color superresolution image, six single-color STED images including centrin, CEP164, CEP290, RPGRIP1L, MKS1, and TCTN2 were first merged using ImageJ (three additive primary colors RGB and three subtractive primary colors CMY). This merged image was then overlapped with the image of TMEM67 (azure color) using ImageJ. To obtain a white-background 7-color superresolution image, a negative process was applied for the whole merged image using ImageJ. To obtain the multicolor superresolution images of IFT88 with TZ/TF/BB proteins, centrin, CEP164, CEP290, RPGRIP1L, TMEM67, and IFT88 were merged using ImageJ with red-hot color assigned for IFT88 images.

## Additional Information

**How to cite this article**: Tony Yang, T. *et al.* Superresolution Pattern Recognition Reveals the Architectural Map of the Ciliary Transition Zone. *Sci. Rep.* **5**, 14096; doi: 10.1038/srep14096 (2015).

## Supplementary Material

Supplementary Information
