# Supplementary material for: Superresolution Pattern Recognition Reveals the Architectural Map of the Ciliary Transition Zone
Source: Sci Rep. 2015 Sep 14;5:14096. doi: 10.1038/srep14096 (PMC4568515; doi:10.1038/srep14096)
Supplement: Supplementary Information [file srep14096-s1.pdf]

Supplementary Information

Superresolution Pattern Recognition Reveals the Architectural Map of the Ciliary Transition Zone

T. Tony Yang[a], Jimmy Su[b], Won-Jing Wang[c], Branch Craige[d], George B. Witman[d], Meng-Fu Bryan Tsou[e], and Jung-Chi Liao[a]

[a]Institute of Atomic and Molecular Sciences, Academia Sinica, Taipei, 10617, Taiwan

[b]Department of Biomedical Engineering, Northwestern University, Evanston, IL 60208, USA

[c]Institute of Biochemistry and Molecular Biology, National Yang Ming University, Taipei, 11221, Taiwan

[d]Department of Cell and Developmental Biology, University of Massachusetts Medical School, Worcester, MA, 01655, USA

[e]Cell Biology Program, Memorial Sloan-Kettering Cancer Center, New York, NY 10065, USA

Supplementary Table 1 Averaged characteristic lengths of ciliary proteins illustrating their sizes and positions at the ciliary base

protein \ nm (N)	Lateral width	Axial width	Lateral diameter	Axial distance to Centrin	Axial distance to TCTN2
**CEP290**	**265±19 (7)**	**213±36 (7)**	**-**	**42±16 (7)**	**-**
**RPGRIP1L**	**284±40 (7)**	**183±30 (7)**	**165±8 (5)**	**143±27 (13)**	**-**
**MKS1**	**335±24 (8)**	**213±26 (8)**	**186±21 (7)**	**150±12 (5)**	**-**
**TMEM67**	**374±30 (10)**	**199±20 (10)**	**205±20 (21)**	**143±15 (8)**	**-**
**TCTN2**	**371±29 (8)**	**197±11 (8)**	**227±18 (17)**	**153±13 (7)**	**-**
**CEP164**	**507±61 (15)**	**-**	**373±35 (14)**	**-38±21 (9)**	**-**
**IFT88 (proximal)**	**-**	**-**	**356±24 (26)**	**-**	**188±25 (10)**
**IFT88 (distal)**	**-**	**-**	**162±11 (3)**	**-**	**126±30 (7)**

Supplementary Table 2 Isotopes and immunogens of primary antibodies

Protein	Isotope	Clonality	Company	Product #	Immunogen
IFT88	rabbit IgG	polyclonal	Proteintech	13967-1-AP	human IFT88 (532a.a.-833a.a.)
RPGRIP1L	rabbit IgG	polyclonal	Sigma-Aldrich	HPA039405	human RPGRIP1L (675a.a.-789a.a.)
TCTN2	mouse IgG2a	monoclonal	Abcam	ab119091	human TCTN2 (full length)
MKS1	rabbit IgG	polyclonal	Proteintech	16206-1-AP	human MKS1 (144a.a.-464a.a.)
TMEM67	rabbit IgG	polyclonal	Proteintech	13975-1-AP	human TMEM67 (43a.a.-364a.a.)
CEP290 (N-terminal)	rabbit IgG	polyclonal	custom-raised	n/a	mouse CEP290 (11a.a.-24a.a.)
CEP290 (C-terminal)	rabbit IgG	polyclonal	Abcam	ab84870	human CEP290 (2429a.a.-2479a.a.)
CEP164	rabbit IgG	polyclonal	Novus Biologicals	45330002	human CEP164 (700a.a.-859a.a.)

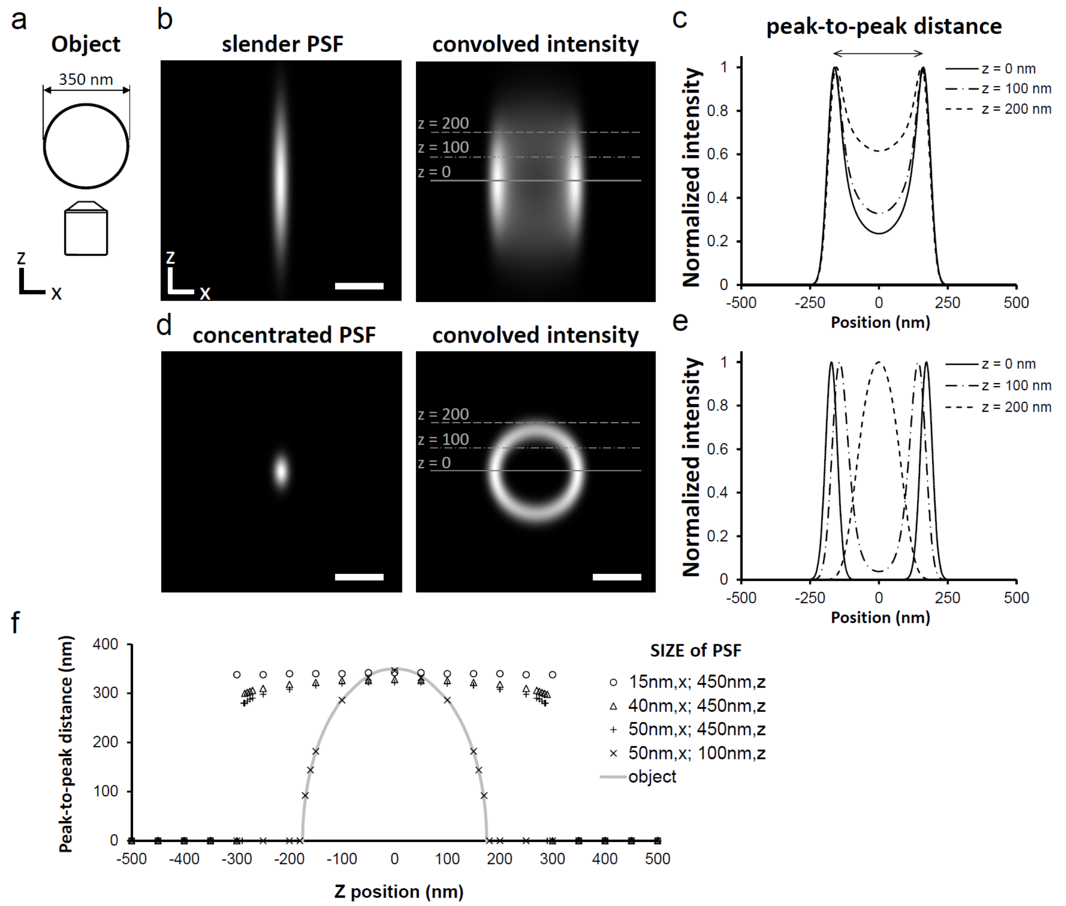

Supplementary Figure 1 Simulation results illustrating the advantage of using the slender PSF of STED in determining radial distributions of ring-shaped proteins. (a) The objective orientation for the imaging of an annular object. (b) The PSF of 2D STED (left) and the simulated convolution intensity (right) of this PSF and an annular object as shown in (a). The convolved image gives a rectangle-like pattern with enhanced signals both in the left and the right edges. (c) Intensity profiles of the convoluted image in (b) at the cross-sections of z = 0, 100, and 200 nm showing the similar side-to-side distance for different focal planes. Thus, the measurement of the ring diameter is not sensitive to the location of the focal plane. (d) A 3D-STED PSF (left) and its simulated convolution intensity for an annular object (right). (e) Intensity profiles of the convoluted image in (d) showing highly focus-dependent patterns with significant changes in the peak-to-peak distance. Thus, the pattern is less recognizable than the one with the slender PSF of 2D STED. (f) Comparison of the

peak-to-peak distances based on 2D-STED and 3D-STED PSFs indicating that 2D-STED PSFs generate much less variations in distances, producing more consistent image patterns. All scale bars, 200 nm.

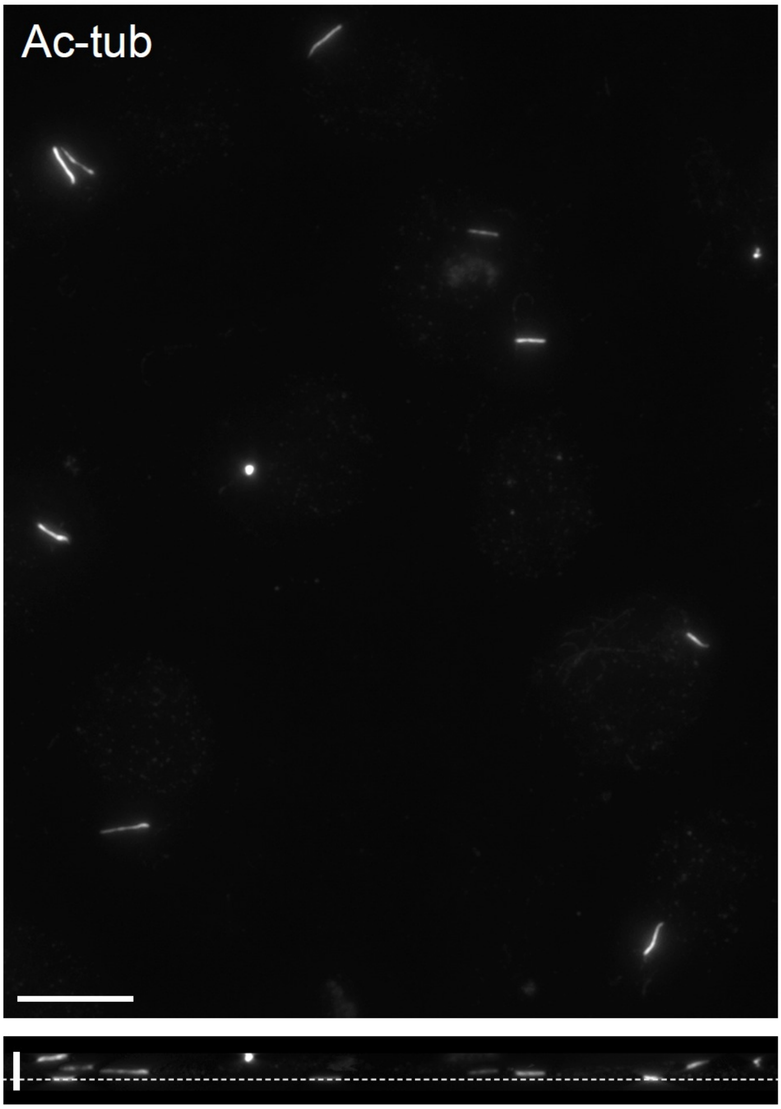

Supplementary Figure 2 Horizontal primary cilia found in RPE-1 cells. (Upper) A stacked image of snapshots at different focal planes. (Lower) A side-view of the 3D stacked image revealed that most of the cilia were oriented horizontally to the culture surface, with the dashed line representing the surface of the coverslip (microscope objective in below). Z step: 200 nm. Scale bars: 10 μm.

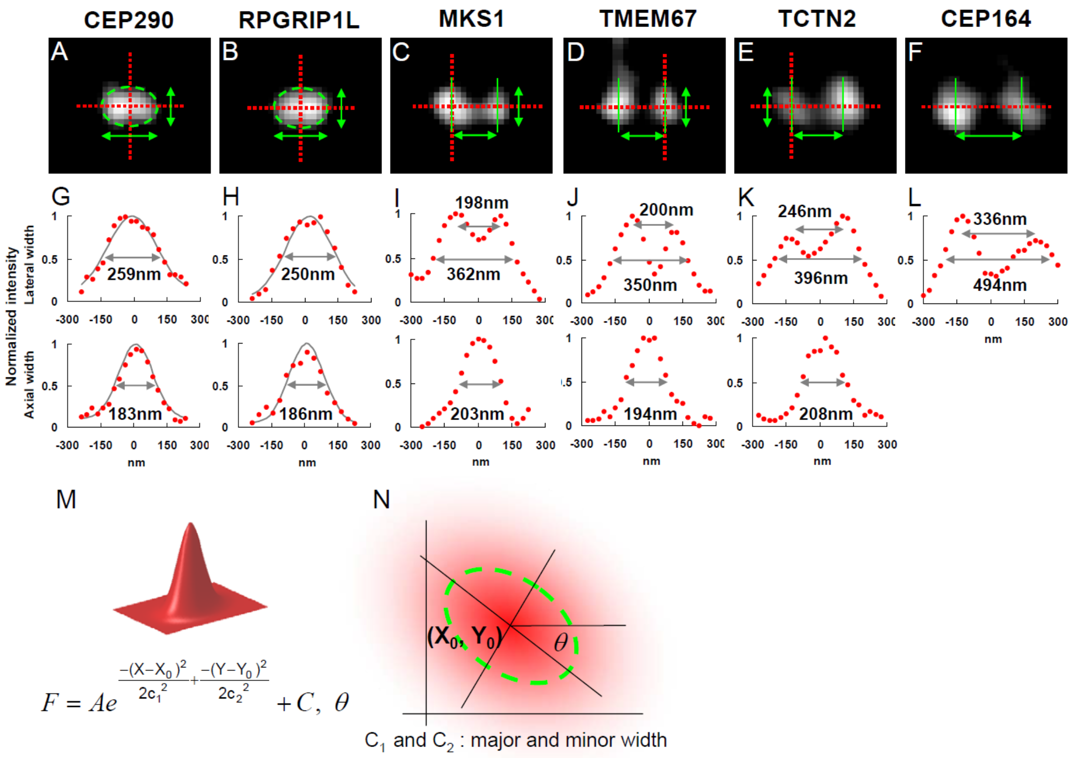

Supplementary Figure 3 Measurement of lateral and axial widths for CEP290, RPGRIP1L, MKS1, TMEM67, TCTN2, and CEP164. (A-F) STED images were measured in lateral (red horizontal line) and axial (red vertical line) directions of the TZ. For CEP290 (A) and RPGRIP1L (B), the data were fitted with a 2D Gaussian function. The green dashed lines show the fitted shape. (C-F) Widths were directly measured along the principal directions of STED images. (G-L) Normalized lateral and axial intensity profiles extracted from images (A-F) along red lines. The peak-to-peak distance was obtained to illustrate the lateral diameter of a ring. (M and N) A rotational 2D Gaussian function was used to determine the width. The FWHMs were obtained by multiplying the coefficients c1 and c2 by the factor $2\sqrt{2\ln 2}$.

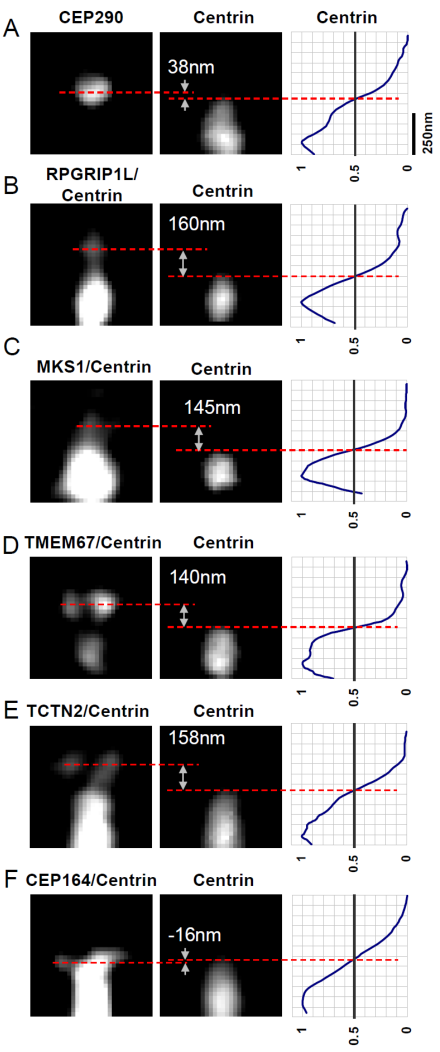

Supplementary Figure 4 The determination of the distal edge of centrin defined as the position where the intensity coincides with the value of the FWHM. (A-F) The distal edge of centrin was first determined by pinpointing the position of the FWHM of signals in each case (right panels). Axial positions of CEP290, RPGRIP1L, MKS1, TMEM67, TCTN2, and CEP164 (left panels) were determined by measuring the distances from the peak intensities of TZ/TF proteins to the locations of the distal edge of centrin (middle panels).

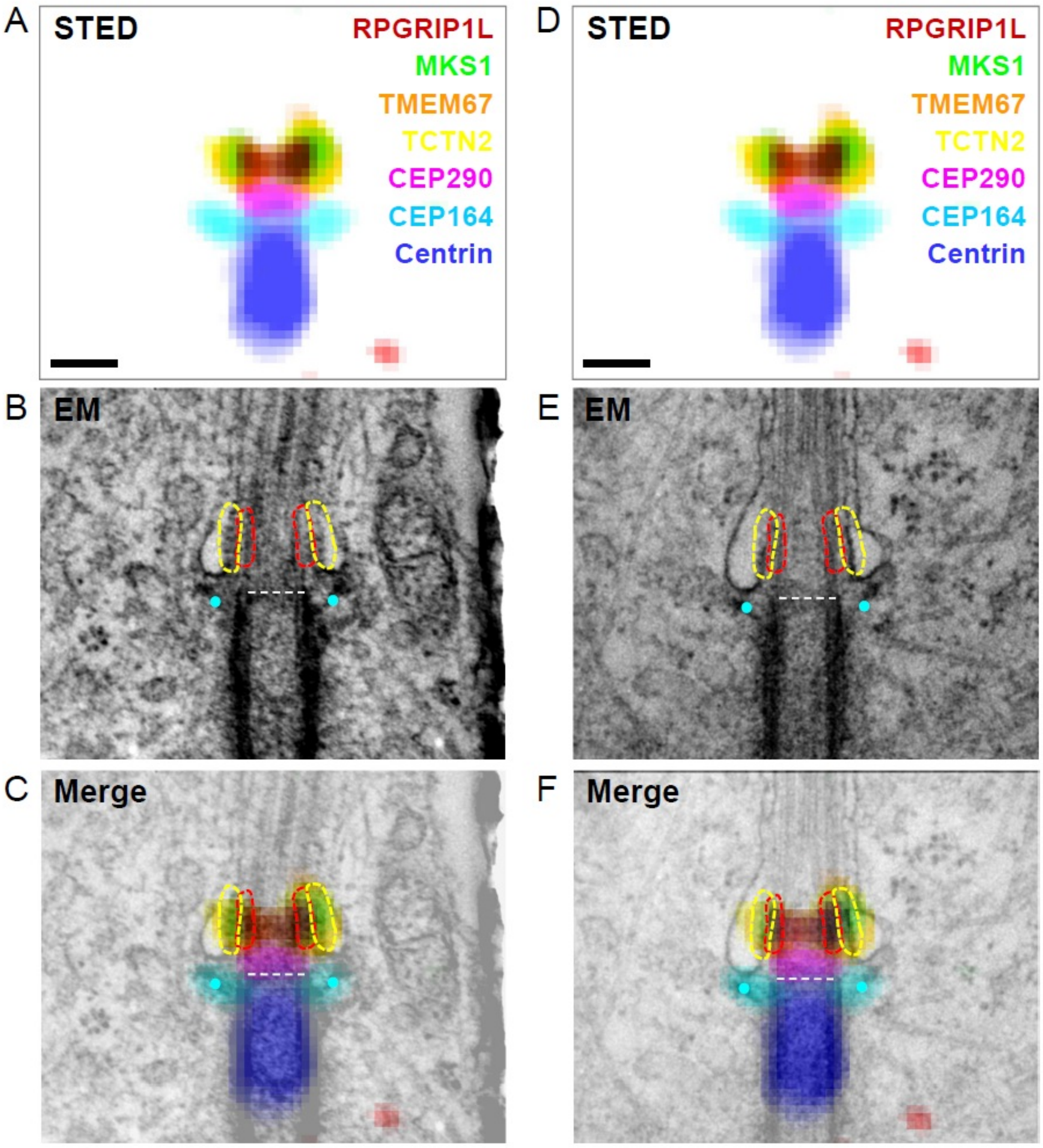

Supplementary Figure 5 Overlapping of multicolor superresolution images of TZ/TF/BB proteins and different EM images revealing their consistent relative localization. (A and D) A 7-color STED image (repeated) demonstrating the relative localization of TZ/TF/BB proteins. (B and E) Two typical EM images of primary cilia in RPE-1 cells in addition to the one shown in Fig. 4, where TFs are marked as cyan dots, areas of microtubule doublets circled with red dashed lines, areas of ciliary membrane covering the ciliary necklace circled with yellow dashed lines, and the distal end of the BB as a white dashed line. (C and F) Merged images from (A) and

(B) or (D) and (E) revealing consistent localization of TZ/TF/BB proteins with respect to the EM ultrastructure. Scale bars: 200 nm.

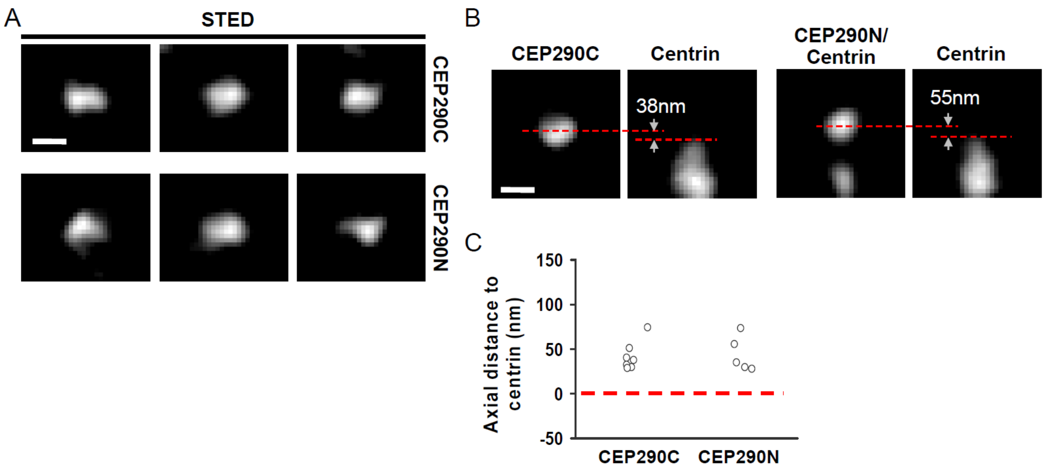

Supplementary Figure 6 Comparison of superresolution images of CEP290 using C-terminal and N-terminal antibodies revealing indistinguishable localization patterns. (A) STED imaging of CEP290 immunostained with C-terminal or N-terminal CEP290 antibodies, named CEP290C and CEP290N, showed similar lateral and axial localizations. (B) The axial positions of CEP290C and CEP290N were determined using the distal edge of centrin as a reference coordinate. Scale bars: 200 nm. (C) Distributions of axial positions of CEP290C and CEP290N were similar. The average distance to centrin is 42±16 nm for CEP290C and 44±20 nm for CEP290N.

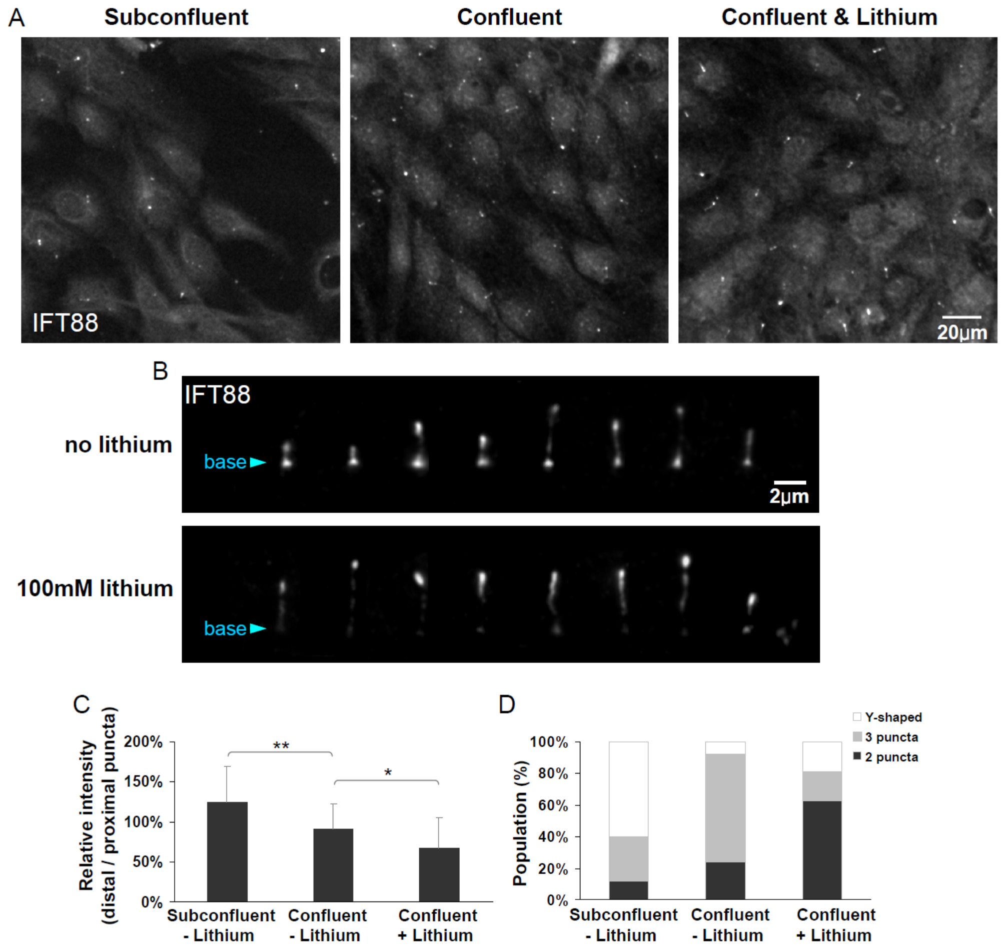

Supplementary Figure 7 Distributions of IFT88 in primary cilia of RPE-1 cells under different growth conditions. (A) Epi-fluorescent images of IFT88 in different conditions. (left) Subconfluent cells (~700 cells/mm^2) had short cilia or had aggregated IFT88 that appeared as dots. (middle) Under confluent conditions (~1000 cells/mm^2), cilia were long and present in most cells. (right) Confluent cells (~1000 cells/mm^2) treated with 100 mM lithium ion (LiCl) for 1 hour before fixation possessed elongated cilia. (B) Lithium effects on IFT88 distribution observed in epi-fluorescent images. Primary cilia with Li$^+$ treatment were longer (lower panel) than those without treatment (upper). IFT88 was evenly localized to the tip and base in untreated cells (upper) while a large proportion of IFT88 moved to the ciliary tip after Li$^+$ stimulation (lower). Arrows show the location of the ciliary base. (C) Superresolution imaging revealed that the relative intensity of the distal punctum to the proximal puncta was affected by ciliary growth conditions. The ratio was close to one in the confluent cells.

In subconfluent cells, the distal punctum was composed of more IFT proteins than the proximal puncta. With Li^+, the intensity of the distal punctum was significantly reduced, leaving a two-puncta pattern. (D) Different population distributions of Y-shaped, three-puncta, and two-puncta patterns in different ciliary growth conditions.

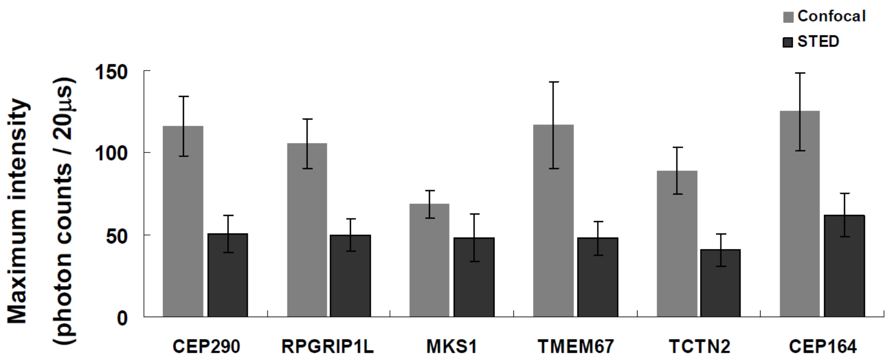

Supplementary Figure 8 Challenges of STED imaging. Intensity comparison of confocal and STED microscopy for TZ/TF proteins revealed the STED imaging signal drops by ~30-60% due to depletion. The Y-axis reports the photon counts collected every 20 μs. Different excitation and depletion laser powers were used for different TZ/TF proteins to reach a comparable and detectable level of photon counts in the STED images.
